# Independent Bottlenecks Characterize Colonization of Systemic Compartments and Gut Lymphoid Tissue by *Salmonella*

**DOI:** 10.1371/journal.ppat.1004270

**Published:** 2014-07-31

**Authors:** Chee Han Lim, Sabrina Voedisch, Benjamin Wahl, Syed Fazle Rouf, Robert Geffers, Mikael Rhen, Oliver Pabst

**Affiliations:** 1 Institute of Immunology, Hannover Medical School, Hannover, Germany; 2 Department of Microbiology, Tumor and Cell Biology, Karolinska Institutet, Stockholm, Sweden; 3 Department of Genome Analytics, Helmholtz Centre for Infection Research, Braunschweig, Germany; 4 Institute of Molecular Medicine, RWTH Aachen University, Aachen, Germany; Stanford University School of Medicine, United States of America

## Abstract

Vaccination represents an important instrument to control typhoid fever in humans and protects mice from lethal infection with mouse pathogenic serovars of *Salmonella* species. Mixed infections with tagged *Salmonella* can be used in combination with probabilistic models to describe the dynamics of the infection process. Here we used mixed oral infections with tagged *Salmonella* strains to identify bottlenecks in the infection process in naïve and vaccinated mice. We established a next generation sequencing based method to characterize the composition of tagged *Salmonella* strains which offers a fast and reliable method to characterise the composition of genome-tagged *Salmonella* strains. We show that initial colonization of *Salmonella* was distinguished by a non-Darwinian selection of few bacteria setting up the infection independently in gut associated lymphoid tissue and systemic compartments. Colonization of Peyer's patches fuels the sustained spread of bacteria into mesenteric lymph nodes via dendritic cells. In contrast, infection of liver and spleen originated from an independent pool of bacteria. Vaccination only moderately reduced invasion of Peyer's patches but potently uncoupled bacterial populations present in different systemic compartments. Our data indicate that vaccination differentially skews the capacity of *Salmonella* to colonize systemic and gut immune compartments and provide a framework for the further dissection of infection dynamics.

## Author Summary

Pathogens have evolved strategies to invade, replicate and spread within their hosts. On the contrary, vertebrates have developed sophisticated immune defence mechanisms that limit, and ideally clear, the infection. This dynamic interplay between host and pathogens determines the course of the infection and the development of clinical disease. Knowledge on particularly vulnerable steps in the infection process, i.e. the “Achilles heel” of a pathogen, may guide the development of anti-infective therapies and vaccines. However, for most pathogens we lack detailed information on the dynamics of the infection process. Here we determined bottlenecks, i.e. critical steps during pathogen invasion and spread, after oral *Salmonella* infection in non-manipulated and vaccinated mice. We infected mice with mixtures of tagged *Salmonella* strains and analysed the strain composition in different compartments by high throughput sequencing. This information allowed us to estimate the number of *Salmonella* invading a given tissue and to describe routes of pathogen dissemination. We show that vaccination only modestly reduces invasion of intestinal lymphoid tissue but had a profound effect on the spread of *Salmonella* to systemic compartments.

## Introduction

While infections with *Salmonella enterica* serovar Typhi and Paratyphi are estimated to affect some 27 million individuals each year [1] nontyphoidal strains of *Salmonella* can also cause life-threatening invasive disease, in particular in immunocompromised patients and by selected *Salmonella* lineages in African countries [2]. Infection with *Salmonella* typically occurs upon consumption of contaminated water or food and thus safe water supply is the best measure to control typhoid and paratyphoid fever [3]. Consequently, due to lack of proper sanitation in developing countries, vaccines remain an important instrument to control typhoid fever [4].

At present two licensed typhoid fever vaccines are available, an injectable capsular polysaccharide vaccine and an orally applied live attenuated bacterial mutant. Parenteral as well as oral vaccines are well tolerated and similarly effective. Still, parenteral and oral vaccines differ with respect to the quality of the induced immune response. Oral vaccines in first instance target gut associated lymphoid tissue (GALT), foremost Peyer's patches (PP), and therein induce a strong gut-directed secretory Ig (SIg) A response [5]. In contrast, parenteral vaccination does not trigger PP and results in comparably weak SIgA responses. The functional relevance of this difference remains uncertain. Intuitively, oral vaccines that recapitulate the natural route of infection might be considered superior compared to the more artificial parenteral route of immunization. Indeed, early studies using mice carrying hybridoma tumors producing IgA directed against a surface epitope of *Salmonella* demonstrated protection after oral but not systemic *Salmonella* infection [6]. Along the same line, infection with a self-limiting invasive *Salmonella* strain yielded high levels of SIgA in the intestine and conferred protection against challenge with fully virulent *Salmonella*, whereas a non-invasive variant that failed to elicit strong SIgA responses did not provide protective immunity [7]. These observations suggest that vaccine-induced SIgA exerts a substantial protective effect at the mucosal surface. Still this interpretation contrasts with other reports. Oral infection with an attenuated *Salmonella* strain protected polymeric Ig receptor (pIgR)-deficient mice that fail to transport and secrete Ig into the gut lumen [8], against secondary infection. Similarly, mice incapable of producing class switched Ig, were protected against infection with fully virulent *Salmonella* by immunization with attenuated *Salmonella* [9].

In humans, a couple of well controlled studies documented antibody and cell-mediated immune responses after natural *Salmonella* infection and vaccination (reviewed in [10]). High serum Immunoglobulin (Ig) levels against *Salmonella* antigens correlated with better protection [11] and antibodies protect against bacteremia caused by non-typhoidal *Salmonella* strains [2], [12]. Still, since *Salmonella* mostly resides within host cells, cell-mediated immune responses are considered to take the lion share in limiting the progression of typhoid infection.

The difficulty to gauge the contribution of different immune defense mechanisms such as cell-mediated versus humoral immunity to the protection against *Salmonella* is linked to limited insight into the dynamics of the infection process. Bacterial loads are commonly described as colony forming unit (CFU), i.e. the number of bacteria recovered under a given condition. In combination with kinetic studies, the CFU adequately describes overall changes in bacterial numbers in a given organ. Still, changes in CFU do not allow disentangling distinct aspects of the infection process such as bacterial growth and death rates, immunological activity, pathogen dissemination and others. However, access to such information could identify critical steps in the infection process and guide the development of vaccines and anti-infective therapies. One way to overcome the limitations of classical infection experiments is to perform co-infections with tagged pathogens and to apply probabilistic models. Such approaches offer unprecedented insights into the dynamics of bacterial populations and may change fundamentally our understanding of bacterial infections [13], [14]. However, so far the potential of such approaches has not been fully exploited. Pioneering studies used PCR or array hybridization to identify the tagged pathogens [15]–[18]. However, allocation of tagged pathogens by PCR or hybridization is a tedious process that seriously limits efficiency and resolution of these approaches. Here we used next-generation sequencing (NGS) of nucleotide-tagged *Salmonella* to identify bottlenecks in the infection process in naïve and vaccinated mice. We exploit stochastic variations in the presence of individual sequence-tagged strains to estimate the number of *Salmonella* setting up the infection and compare the composition of tags in different compartments to describe their relatedness and deduce routes of pathogen dissemination. We reveal vaccination-sensitive and insensitive steps of the infection process in otherwise non-manipulated wild type mice. Our results suggest that vaccination has only a modest effect on initial colonization but potently restricts pathogen spread in systemic compartments.

## Results

### Construction and validation of genome-tagged clones of *S.* Typhimurium

Vaccination may limit initial invasion, affect subsequent growth of bacteria and/or affect pathogen dissemination. To describe the dynamics of the *Salmonella* infection process in mice, we performed infections with mixed inocula of genome-tagged strains of *Salmonella enterica* serovar Typhimurium (*S.* Typhimurium). For creating such tagged strains, primers carrying a random nucleotide sequence were used to generate genomic integrations, disrupting the endogenous *proV* gene and introducing an artificial stop codon. A total of 23 strains carrying individual sequence tags was selected for easy-to-discriminate tag sequences and used for further experiments.

The *proV* gene is one of three redundant genes under the *proU* operon, which encodes a glycine-betaine/L-proline binding transport system during high osmolarity situation. Genetic disruption of *proV* is thought to have no effect on *Salmonella* virulence [19]. Still, before applying our tagged collection of strains in infection experiments, we compared the fitness of the wild type and its isogenic genome-tagged strains for their virulence in C57/BL6 mice. After either oral or intra peritoneal infection the parental *S.* Typhimurium strain and its tagged variants revealed comparable bacterial loads in the mesenteric lymph nodes (mLN), PP, livers and spleens (Fig. S1). This confirmed that disruption of the *proV* gene did indeed not affect colonization or expansion of *S.* Typhimurium *in vivo*. According to a previous suggestion for naming such collections of strains [17], we refer to our library of tagged strains as wild type isogenic tagged strains of *S.* Typhimurium, or WITS.

### Non-Darwinian selection of *S.* Typhimurium during infection of intestinal and systemic tissues

**Figure 1:**
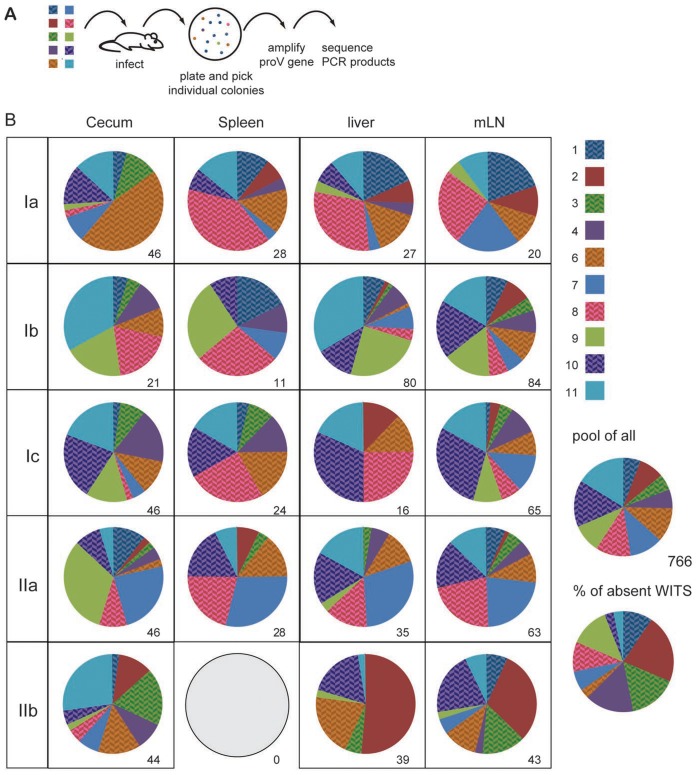
Individual WITS disproportionally contribute to the infection. (A) C57BL/6 mice were orally infected with a mixture of 10 individually tagged WITS. Two days after infection mice were sacrificed and cecal content, spleen, liver and mLN plated. Individual colonies were picked and sequenced. (B) Pie diagrams depict the contribution of the 10 different WITS in each compartment and numbers indicate the number of colonies analysed. WITS clone 5 has intentionally been excluded because this clone did not grow well in this particular experiment. The five mice depicted were infected in two independent experiments (first experiment Ia, Ib, Ic and second experiment IIa and IIb). For the characterization of the WITS composition, we selected five out of eight mice infected in two independent experiments, which showed colonisation levels fairly close to the median CFU of the entire group. CFU for the five mice included in this figure were as follows (median (minimum - maximum)): spleen: 104 (42–1080); liver: 305 (110–635) and mLN: 3720 (1880–5260). All WITS appeared fairly evenly represented when all 766 sequences were pooled and all 10 WITS were occasionally absent from individual compartments analysed.

To increase the recovery rate of a given WITS, we first used an equal mixture of 10 instead of 23 WITS to orally infect C57BL/6 mice. Two days after infection, cecum contents and minced Peyer's patches (PP), mesenteric lymph nodes (mLN), livers and spleens were plated. Individual colonies were picked up, the tagged *proV* gene was amplified by PCR and sequenced by Sanger technology. When analysing the representation of the individual WITS, we observed that within single compartments, some WITS were strongly over- or underrepresented, and some WITS were entirely undetectable (Fig. 1). Since we analysed only a low numbers of colonies, we evaluated whether absence of some WITS might simply reflect sampling and analysing a low number of colonies. However, *in silico* simulation suggested that most experimentally observed WITS compositions were highly unlikely to occur by chance (data not shown). This indicated that already 2 days post challenge selection processes profoundly shaped the bacterial population. Moreover, we observed striking differences between compartments, and individual WITS dominating one particular compartment could be less frequent or undetectable in others (Fig. 1). In particular, the representation of individual WITS as observed in the cecum did not show a congruent match to the situation observed in liver, spleen or mLN of the same mouse. Similarly, when comparing the distribution of WITS among liver, spleen and mLN, no consistent representation of WITS was found. This suggested that independent selection processes occur within the intestinal lumen and beyond the gut. Notably, when we pooled sequences from all infected animals and compartments, we observed a fairly equal contribution of the 10 WITS. Moreover, all 10 WITS were represented in most compartments analysed and each of the WITS was absent in some rare cases (Fig. 1). This further confirmed the equal biological properties of the individual WITS.

Over-represented WITS might arise from stochastic variations in the infection process in combination with an overall low number of bacteria initially invading the tissue. Alternatively, over-represented WITS might have acquired beneficial mutations, which allowed them to outgrow other WITS *in vivo* and thereby resulting in a disproportionate representation of individual WITS. To distinguish between these alternatives, we performed sequential infection experiments in 129Sv mice. 129Sv mice, which are more resistant than C57BL/6 mice and survive *S.* Typhimurium infection, were infected with an equal mixture 10 WITS for 60 days. Similar to the findings described in the acute infection, individual WITS were unevenly represented in the mLN 60 days after infection (Fig. S2).

Subsequently, we picked one over- and one under-represented WITS from two chronically infected mice, and used them at a 1∶1 ratio for a second round of infection in naïve 129Sv mice. Two days later, the contribution of both WITS was determined. In one case we observed a scenario opposite to the first round of infection/selection, in another case both WITS contributed roughly equally in the second infection (Fig. S2). Even though we did not perform extensive analysis on larger groups of mice, this results hints that the disproportional contribution of individual WITS might not be caused by acquirement of stable genetic mutations in the bacteria. Instead, a seemingly non-Darwinian selection process appeared to favour or repress individual WITS early after oral infection.

### A framework model for estimating the number of bacteria founding infections

We speculated that the seemingly random presence or absence of WITS within individual host compartments and their disproportional contribution to the bacterial load could be used to describe population dynamics of infecting bacteria. In case of numerous invasion events, i.e. if the number of bacteria entering a given compartment largely exceeds the number of WITS, all WITS should be evenly represented in the tissue. In contrast, our data rather suggested that a few founder bacteria set up the infection, effectively resulting in a heavily skewed representation of WITS and the absence of several WITS in a given compartment. Thus, we propose that determining the frequency of samples that lack one or more WITS allows an estimate of the number of bacteria that initially seed a given compartment and successfully contribute to the observed population. We refer to this number of bacteria that initially invade an organ and initiate a productive infection, as “tissue seeding units”, TSU. To translate experimentally observed frequencies of ‘one or more missing WITS’ into TSU values, i.e. the most probable number of bacteria contributing to the infection, we applied a stochastic model. As input parameters we allowed variable numbers of WITS to be represented at definable proportions. As output parameter the model provides the most likely TSU, i.e. the most likely number of invasive bacteria that corresponds to a given frequency of experimentally observed ‘missing WITS’. Since real life experiments are limited to comparably small sample sizes/numbers of mice, we additionally allowed to define the number of samples. This enables us to determine confidence intervals besides the mean value expected for very large sample sizes.

To calculate a TSU estimate on the basis of the frequencies of ‘one or more missing WITS’, we established a software to determine TSU and confidence intervals. A detailed description of the model and the visual basic application (VBA)-coded macro, which runs in the Microsoft Excel environment, are available as supplementary material.

**Figure 2:**
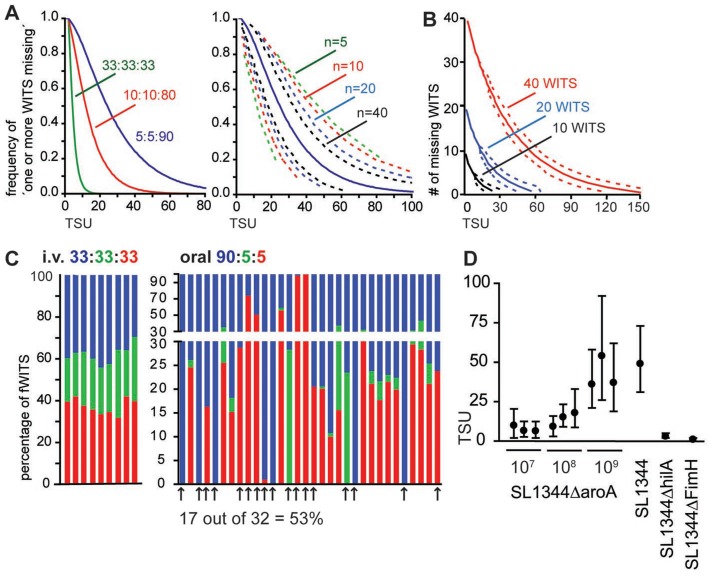
A statistics based method to determine the number of bacteria founding the infection. (A) *In silico* simulation to translate the frequency of ‘one or more missing WITS’ into TSU. Numbers indicate the ratio at which three different WITS have been mixed (left panel) or the number of samples analysed (right panel; plotted for a ratio of 5∶5∶90). Dashed lines indicate 95% confidence intervals. (B) With increasing numbers of WITS, additional information can be gained by considering the number of WITS missing. Lines indicate predicted TSU for 10, 20 and 40 WITS used at equal proportion and one sample analysed. Dashed lines indicate 95% confidence intervals. (C) Representation of three fWITS in PP two days after *i.v.* or oral infection at the indicated ratio. Bars represent the frequency of the color-coded fWITS in individual PP in two (left panel, *i.v*.) or 5 (oral right panel) individual mice. Arrows mark PP that show ‘one or more missing WITS’. (D) TSU determined in PP at day 2 post infection for different infection doses and *Salmonella* strains as indicated. Circles represent the predicted TSU, and whiskers represent 95% confidence intervals for independent experiments.

Mixing the WITS at different proportions allows to shift the range of TSU that can reasonably be determined (Fig. 2A). Irrespective of the number of samples analysed, only a single frequency of ‘one or more missing WITS’ in all samples can be determined and consequently each experiment will yield only a single TSU value. Still, the explanatory power of TSU data depends on the number of samples analysed and confidence intervals associated with a given TSU narrow with increasing numbers of samples (Fig. 2A, right panel). With increasing numbers of WITS, the approach can be further modified to include the information of how many WITS are missing, instead of the ‘one or more missing WITS’. With large numbers of WITS used in the mixed inocula, reasonably robust TSU values can be determined even for individual samples (Fig. 2B).

Picking and sequencing of individual clones is poorly suited to determine the frequency of ‘one or more missing WITS’, because a high number of colonies need to be analysed by a low-throughput method. One straightforward method to reliably determine the frequency of ‘missing WITS’ is provided by fluorescence-tagged bacteria. Characterization of nucleotide-tagged bacteria by saturating sequencing techniques, i.e. when all clones are sequenced, represents a second approach (see below).

To challenge the TSU concept, we performed mixed inocula infections with fluorescent *S.* Typhimurium strains, here referred to as fluorescent WITS (fWITS). To minimize the impact of the fluorescent marker on *Salmonella* virulence, all reporter proteins were expressed under control of an inducible promoter that is transcribed at very low rate *in vivo* but readily inducible *in vitro* by addition of arabinose to the medium. Two days after *i.v.* infection with a mixture of three fWITS, all fWITS were represented in individual PP at fairly similar proportions compared to the inoculum and we did not observe ‘one or more missing WITS’ (Fig. 2C, left panel). Similarly, when an equal mixture of the fWITS was used for oral infection, the vast majority of all PP harboured all fWITS (data not shown). To increase the occurrence of ‘one or more missing WITS’, we used the fWITS at a 90∶5∶5 ratio that compared to the equal mixture is more suitable to observe higher TSU (Fig. 2A). In this setting again all fWITS were represented in pooled PP (data not shown). Yet, the situation changed when individual PP were analysed and similar to our observations with the nucleotide-tagged WITS library (Fig. 1), within single PP the representation of individual fWITS frequently differed from the inoculum. Additionally, at a 90∶5∶5 ratio we observed ‘one or more missing WITS’ in roughly half of all analysed PP, i.e. at least one fluorescent strain could not be detected (Fig. 2C). Notably, for this analysis only samples/plates were considered that showed a sufficient number of colonies, so to exclude false ‘one or more missing WITS’ observations with a p value of 0.05. For oral infections with 10^9^ *Salmonella* cells, experimentally observed frequencies of ‘one or more missing WITS’ translated into 42 TSU per PP (confidence interval 23–71). Reducing the infection dose from 10^9^ to 10^8^ and 10^7^ attenuated *Salmonella* resulted in a reduction of the TSU per PP (Fig. 2D). However, the number of bacteria entering the PP did not decline proportionally with the infection dose. Similar TSU were observed at day 2 in PP of C57BL/6 and 129Sv mice as well as comparing SL1344 parental strain to the attenuated SL1344Δ*aroA* strain (Fig. 2D and data not shown). This indicated that differences in host susceptibility to *S.* Typhimurium had no detectable effect on TSU. In contrast a *Salmonella* pathogenicity island-1 (SPI-1)-deficient strain (SL1344Δ*hilA*) or a *Salmonella* strain lacking a fimbrial subunit (SL1344Δ*fimH*) showed very low TSU (Fig. 2D). These results are consistent with the known function of SPI-1 in tissue invasion [20] and of FimH in binding to M cells [21].

**Figure 3:**
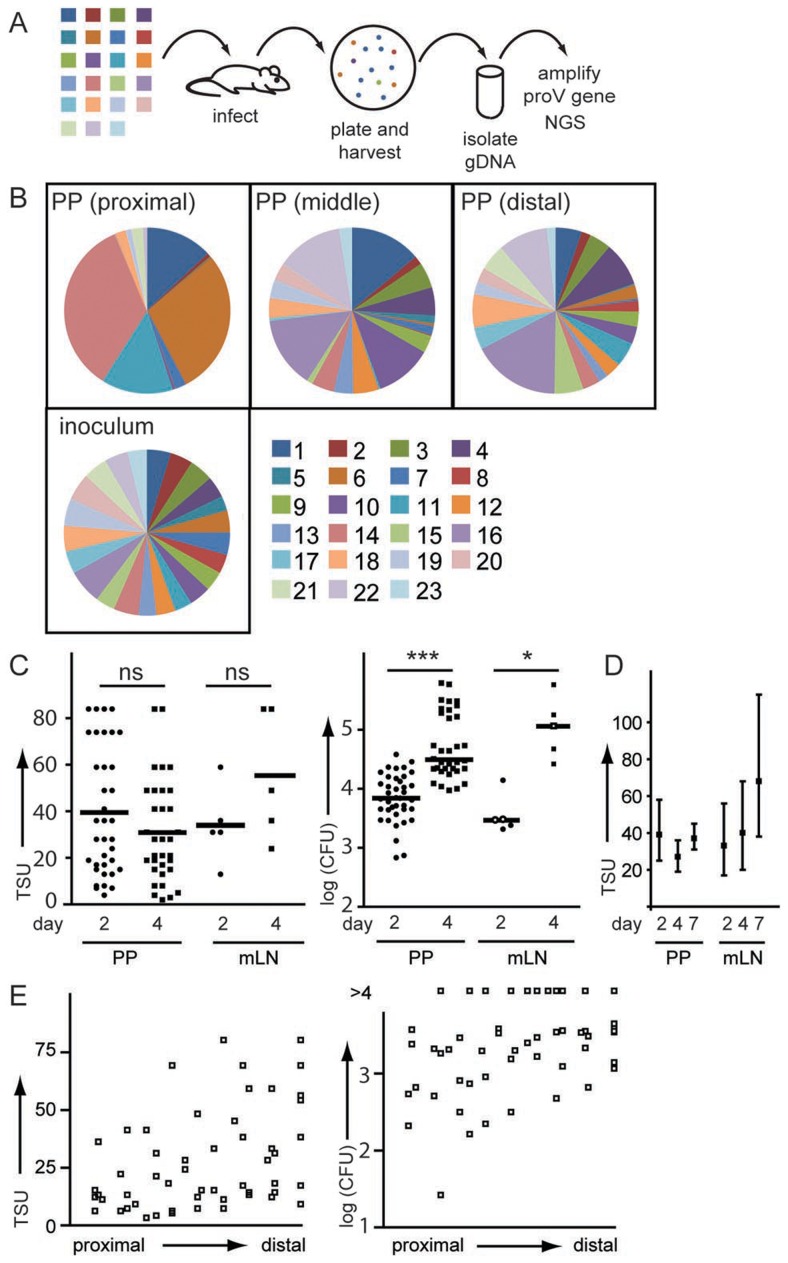
Individual PP are colonized by an independent pool of *S.* Typhimurium. (A) Mice were orally infected with an equal mixture of 23 WITS. 2 days post infection organs were plated and all colonies harvested by washing of the plates with PBS. Bacteria were pelleted, gDNA isolated and the *proV* gene amplified. Amplicons were sequenced by NGS. (B) Pie diagrams depict representative examples of WITS composition in proximal, middle and distal PP (C) TSU and CFU were determined for individual PP and mLN 2 and 4 days post infection. Symbols represent individual organs pooled from three experiments. (D) TSU were determined 2, 4 and 7 days post infection with attenuated SL1344Δ*aroA* fWITS. Mean TSU and confidence interval were determined for a total of 20 infected mice per time point. (E) TSU and CFU were plotted according to their position along the small intestine.

While already the use of two WITS allows determining TSU, the practicability of this approach is limited because robust values can only be obtained for comparably large sample sizes/numbers of mice (Fig. 2A). We therefore performed mixed infections with our nucleotide-tagged WITS library and used NGS to determine the representation of individual WITS. Mice were orally infected with an equal mixture of 23 WITS. 2 or 4 days post infection PP were minced and plated. Instead of picking individual clones, we recovered all grown colonies, extracted genomic DNA, bulk amplified the tagged *proV* gene by PCR and analysed the amplicons by NGS (Fig. 3A). To ensure sufficient coverage for each sample, at least ten-fold more sequences than ‘input colonies’ present on the plate were analysed. Technical replicates showed that PCR amplification and NGS were highly reproducible and did not affect our results (Fig. S3). Consistent with our previous observations, we observed a disproportional representation of WITS in single PP and frequently one or more WITS were absent (Fig. 3B). Since NGS analysis of WITS composition (Fig. 3) and characterisation of WITS composition by colony picking and Sanger sequencing (Fig. 1) both show similar patterns, including strongly overrepresented as well as missing WITS, we suggest that these features of the WITS composition are unlikely to be caused by PCR bias or during NGS. TSU determined by NGS for individual PP stayed well within the TSU range predicted on the basis of fWITS. Moreover, the TSU predicted for PP did not depend on the day of analysis, i.e. 2 and 4 days post infection we observed comparable TSU, even though CFU were much higher at day 4 compared to day 2 post infection (Fig. 3C). In addition, the higher resolution offered by a more complex WITS library allowed to estimate TSU for mLN = 25 (13–36), spleen = 24 (1–46) and liver = 36 (8–65).

Considering that each mouse has 8–12 PP, 2 days post infection the overall number of *S.* Typhimurium entering PP by far exceeds the number of bacteria reaching mLN and/or liver and spleen. However, we also noted that 4 days after infection TSU in mLN tended to be higher than at day 2 (Fig. 3C), whereas no such difference was observed in PP (Fig. 3C). Such increase in TSU observed in mLN might indicate sustained dissemination of *Salmonella* from the gut into mLN.

Since *Salmonella*-caused lethality prevented the analysis of later time points, we used the attenuated SL1344Δ*aroA* fWITS to compare TSU at 2, 4 and 7 days post infection. Indeed we observed that comparing day 2 and 7 post infection, TSU were higher in mLN but not in PP (Fig. 3D). Comparing TSU for different PP along the axis of the small intestine, we noted a tendency to higher TSU in distal compared to proximal PP that also reflected in moderately higher CFU (Fig. 3E). In aggregate, these observations indicated that firstly, after primary infection between 2 and 4 days post infection there is no on-going invasion of PP from luminal *Salmonella* and secondly, no ‘new holes’ appeared in the WITS pool, i.e. WITS present at day 2 were also present at day 4. This observation emphasizes that TSU do not correlate with CFU but represent an independent measure to describe the infection.

### Tracing routes of *S.* Typhimurium dissemination by WITS sequencing

Besides determining holes in the WITS pool, NGS characterization of WITS populations can be used to track *S.* Typhimurium dissemination routes. Compartments successively colonized from a joined set of bacteria should show a similar representation of WITS. In contrast, a dissimilar composition of WITS in two compartments rather indicates their independent colonization.

**Figure 4:**
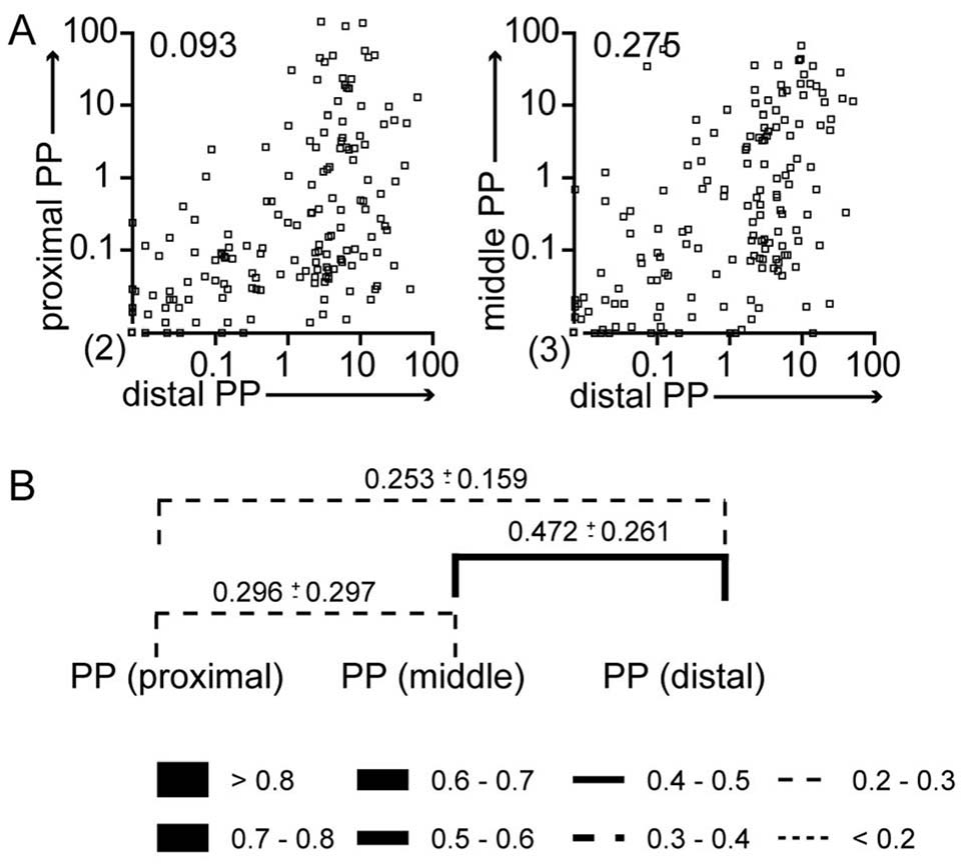
WITS similarity reflects routes of *S.* Typhimurium dissemination. Mice were treated as described in Fig. 3A. (A) The frequency of individual WITS in proximal and distal PP (left panel) or middle and distal PP (right panel) was compared. Numbers indicate Pearson correlation coefficients. Symbols represent individual PP pooled from 3 independent experiments. (B) Similarity of WITS representation was calculated as Morisita-Horn index. Lines indicate pairwise comparisons. Numbers indicate the MHI±SD. Line thickness/style depicts average MHI as indicated. Pearson correlation coefficients are higher comparing middle to distal compared to proximal to middle PP. Still, no significance was detected using 2-way ANOVA.

Two days after oral infection the WITS representation in individual PP appeared random, i.e. the frequency of a given WITS present in one PP was not predictive for the frequency of the respective WITS in another PP of the same mouse (Fig. 4B). To systematically compare the WITS composition in different compartments, we performed Pearson correlation analysis and calculated Morisita-Horn indices (MHI) to describe population similarity. For Pearson correlation analysis the frequency of each WITS in one compartment was plotted versus its frequency in a second compartment (Fig. 4A and S4). Consistent with the visual impression, comparison of proximal PP to distal PP showed a very low correlation coefficient and likewise only slightly stronger correlations were observed comparing middle to distal PP. Still, the Pearson's correlation coefficient is sensitive to skewed distributions and outliers that are an inherent feature of the WITS data discussed here. We therefore used the Morisita-Horn index (MHI) to quantify similarities in the WITS composition seen in different compartments. The MHI gives weight to unique species such as missing WITS and provides a weighted measure of population similarity. A MHI of one identifies identical populations, whereas a MHI of zero reflects entirely dissimilar populations. Comparing the WITS composition between different PP low MHI were observed for the comparison of proximal to distal and slightly higher MHI were observed for middle to distal PP. In sum, these results indicate that *S.* Typhimurium invading PP did not undergo strong selection in the gut lumen and colonization of individual PP is an independent process.

### Vaccination reduces invasion by *Salmonella* and skews dissemination routes

**Figure 5:**
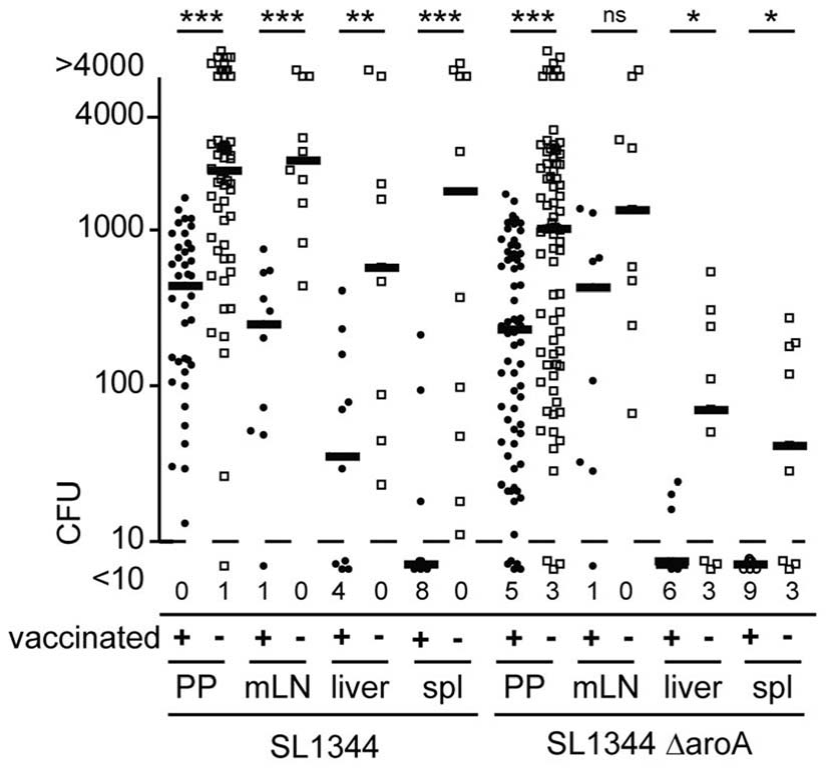
Infected organs show different levels of vaccination-mediated protection. C57BL/6 mice were orally vaccinated with attenuated SL1344Δ*aro*A *Salmonella* Typhimurium. 40–50 day later vaccinated mice and age-matched controls were orally infected with 1×10^9^ of either SL1344 or SL1344Δ*aro*A. CFU were determined after two days for single PP, mLN, liver and spleen (spl). Symbols indicate individual organs and bold lines median CFU from 9 or more mice pooled from 2 independent experiments. Numbers indicate organs displaying less than 10 CFU.

We next characterized *Salmonella* dissemination and invasion frequencies in *S.* Typhimurium *aroA* vaccinated mice as compared to non-vaccinated age matched controls. Mice were vaccinated with SL1344Δ*aroA* 40–50 days before secondary infection with either a fully virulent SL1344 or an SL1344Δ*aroA* strain. CFU in target organs were consistently higher in naïve mice as compared to mice vaccinated with the attenuated *aroA*-deficient *S.* Typhimurium strain. However, the level of protection as seen as fold reduction in CFU differed for the various compartments (Fig. 5). In particular, after challenge with the virulent SL1344 strain, the drop in CFU observed in vaccinated compared to non-vaccinated mice was less pronounced in PP compared to mLN and spleen. This indicates that vaccination did not protect all compartments to the same extent.

**Figure 6:**
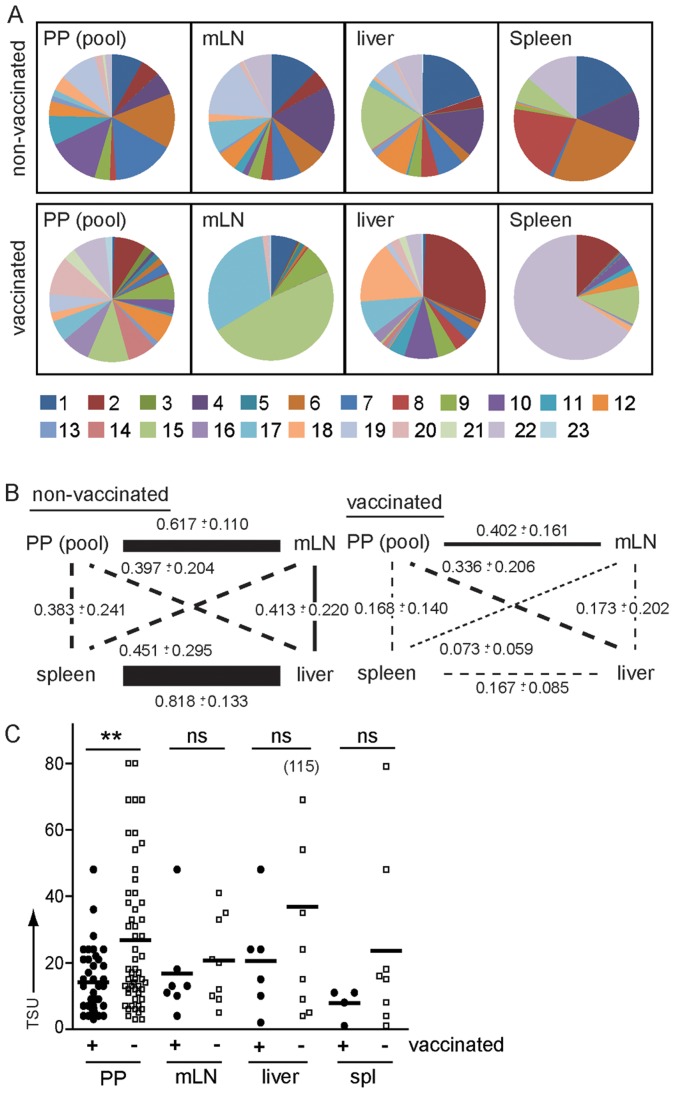
Vaccination modulates routes of *S.* Typhimurium dissemination. Mice were vaccinated with a SL1344Δ*aroA* strain 40–50 days or left untreated before infection with the WITS library. WITS composition was determined as described in Fig. 3A. (A) Pie diagrams depict representative examples of WITS composition in pooled PP, mLN, liver and spleen of one non-vaccinated and one vaccinated mouse. (B) Similarity of WITS composition was compared between various compartments. Numbers indicate MHI for the various comparisons and line width/style depicts MHI as indicated in Fig. 4B. 2-way ANOVA reveals that the effect of vaccination on the similarity between compartments (determined by MHI) is significant (p<0.001). Mann-Whitney t test revealed significant effects on distinct compartments. Vaccination significantly affected similarity of the WITS composition between PP and mLN (p = 0.016), mLN and liver (p = 0.026), mLN and spleen (p = 0.016) and liver and spleen (p = 0.004). Vaccination had no significant effect on the similarity in WITS composition observed for the comparison of PP and liver or PP and spleen. (C) Vaccination only moderately reduced invasion of PP. Symbols indicate TSU predicted for individual PP, mLN, liver and spleen of non-vaccinated and vaccinated mice pooled from 3 or more independent experiments. Horizontal bars indicate the mean.

When infecting vaccinated and non-vaccinated mice with the WITS library and probing proportions of WITS 2 days post infection, uneven WITS composition and holes in the WITS library were observed in mLN, liver and spleen of both vaccinated and non-vaccinated mice (Fig. 6A and S4). When comparing the WITS composition between various compartments in non-vaccinated mice, the highest MHI were observed at PP to mLN and liver to spleen comparisons (Fig. 6B and S4). In contrast, the WITS composition in PP and mLN was largely dissimilar to the composition noted for the liver and spleen. This indicates that *S.* Typhimurium colonizing systemic compartments did not originate from PP or mLN. Consistently, we observed that depletion of CD11c-expressing cells resulted in decreased TSU in mLN but not in liver and spleen (Fig. S5). Depletion of CD11c-expressing cells foremost affects dendritic cell (DC) numbers. Thus, reduced TSU after DC depletion in mLN but not systemic compartments supports our previous suggestion that dissemination from gut/PP to mLN but not to liver and spleen relies on DC-mediated transport of *Salmonella* [22]. Instead, colonization of liver and spleen seems to originate from an independent pool of bacteria.

In contrast to non-vaccinated mice, in orally vaccinated mice similarity of WITS composition was reduced for all compartments. Still the strongest effect was apparent for the comparison of liver and spleen, whereas the association of PP and mLN was less affected (Fig. 6B). This indicates that vaccination does not only affect killing of *Salmonella*-infected cells by cell-mediated immunity but also alters *Salmonella* migratory routes, potentially by the action of vaccination-induced *Salmonella*-specific antibodies. Antibodies might be particularly effective in uncoupling colonization of liver and spleen, whereas the lymphogenic spread of *Salmonella* from PP to mLN might be less sensitive to antibody-mediated protection.

Besides serum antibodies, oral and to a lesser extent parenteral vaccination induces SIg, predominantly SIgA, secreted into the gut lumen. SIg may shield host tissues from pathogens but also modulate sampling of SIgA-bound antigen [23]. The invasion frequency of *Salmonella* into PP of vaccinated mice was significantly reduced compared to non-vaccinated age-matched controls but still substantial numbers of bacteria were able to invade (Fig. 6C). This indicates that even though vaccination is insufficient to completely shield PP from invasion, vaccinated mice will still be confronted with a reduced number of *Salmonella* after oral infection. This difference might be decisive in natural settings that, compared to the very high number of *Salmonella* used in experimental infections, typically are distinguished by lower numbers of *Salmonella* taken up with the diet – an observation that warrants to be considered in the development of novel anti-*Salmonella* vaccines.

## Discussion

Within-host changes in bacterial loads are influenced by numerous factors, including bacterial replication, dissemination and killing by the host. To understand how these and other factors determine bacterial loads is critical to develop anti-infective strategies. Thus, sophisticated technical approaches have been established to dissect the impact of distinct bacterial and host factor on the course of bacterial infections. One approach to distinguish bacterial replication and killing of bacteria used non-replicating elements (reviewed in [24]). Similar to this study, other reports used mixed infection with tagged bacteria. Fluorescently tagged *Yersiniae enterocolotica* [25] and oligonucleotide-tagged *Y. pseudotuberculosis* [15] have been used to describe the dynamics of systemic infection in mice. A chief finding from such studies is that systemic infection may originate from clonal expansion of only few individual bacteria residing in infection foci [25]. While being chiefly an intracellular pathogen during the systemic phase of the murine infection, microscopic studies on *S.* Typhimurium-infected murine livers have also revealed individual infection foci of infected macrophages [26]. Likewise, seminal studies using two or three genetically tagged *S. enterica* strains in combination further imply that also productive infection is initiated from selected infective units rather than through an uniform progression of the infection from the mixed inoculum [27].

Manipulation of host and pathogen factors can be expected to further alter the outcome of infection dynamics, and to provide fundamental insights into infection pathogenesis. However, the approaches listed above may fall short of describing the fine architecture of pathogen dynamics and relevant information required for the rational design of anti-infective therapies [14]. Here we used mixed inocula of tagged genetically tagged *S.* Typhimurium (wild type isogenic tagged strains; WITS), identifiable by rapid next-generation DNA sequencing, to detail *in vivo* population dynamics in naïve and vaccinated mice after oral infection.

Following oral infections with a WITS library, we observed a highly uneven representation of WITS in target organs and frequent holes in the WITS library. NGS offered an affordable and fast method to characterize such WITS compositions. In this study typically more than 10-fold more sequences than bacteria present in the sample were analysed – still depending on total CFU and the number of different WITS, far fewer sequences are sufficient to reliably describe the WITS representation in target organs. We propose firstly that holes in the WITS pool can be used to estimate the number of bacteria productively seeding a given compartment; and secondly, that comparison of WITS representation between samples allows describing routes of *Salmonella* dissemination.

Both properties of the WITS pool, ‘holes’ and skewed representation of individual WITS, seem interconnected and a consequence of an overall low number of *S.* Typhimurium initially seeding target organs. We assume that detectable WITS regularly expand from single bacteria and subsequently grow up to either overrepresented WITS clones or show low net growth and eventually contribute to the overall bacterial load to a much lower degree. Thus, the particular nature of the infected host cell might determine the contribution of the respective bacteria to the overall bacterial population. This observation is consistent with the previously described ‘dormant’ *Salmonella*, bacteria which are infecting host cells but not undergoing direct replication [28]. Consequently, we did not use the frequency of a given WITS to estimate invasion frequencies but instead only considered absence/presence of a given WITS.

The occurrence of ‘holes’ in the WITS representation can be used to estimate the number of bacteria that initially seeded a compartment. We refer to such values as tissue seeding units – TSU. TSU for PP, mLN, liver and spleen were typically below 50, i.e. less than 50 founder bacteria set up the infection. Considering the number of 10^9^ *Salmonella* used for oral infection these values seem surprisingly low. Yet, similar observations were recently reported in the “streptomycin model” of *Salmonella* infection. In this model despite presence of 10^9^ *Salmonella* in the cecum only about 300 *Salmonella* reached the draining lymph nodes per day [18].

TSU were similar for attenuated (Δ*aroA*) and wild type *S.* Typhimurium as well as in susceptible C57BL/6 mice and more resistant 129Sv mice (data not shown). This indicates that metabolic attenuation as well as relevant host factors affecting the killing of incoming *Salmonella* did not affect the number of bacteria initially breaching the gut barrier. Still the TSU is based on detectable bacteria in a specific organ and we cannot rule out that higher numbers of bacteria initially entered host tissues but are undetectable even in susceptible hosts. Irrespectively, TSU measurement allows uncoupling initial invasion from later steps of the infection process and reveals that entry from the gut lumen into host tissues represents the first bottleneck in oral infections that is overcome by only few bacteria.

Comparing the TSU at different time points after infection provides information on the spreading of the infection over time, i.e. increasing TSU with time might indicate sustained colonization of a given compartment. Such effect could be observed in mLN but not liver and spleen, i.e. in mLN TSU at later time points were higher compared to day 2 after infection. Increasing TSU in mLN over time might be fuelled by infected PP, which in sum encompassed all 23 WITS present in the library. *Salmonella* can be transported from the gut to mLN within DC. Colonization of the mLN is reduced in CCR7-deficient mice that display impaired migration of DC [22], [29] and also in the streptomycin model about 10-fold reduced rates of *Salmonella* entering draining lymph nodes were observed [18]. Here we show that depletion of DC reduces the number of *Salmonella* entering mLN but not liver and spleen and TSU in mLN but not systemic compartment gradually increased during the infection.

A higher resolution of *S.* Typhimurium dissemination within the host could be achieved by comparing the WITS frequencies between different compartments. Comparison of WITS representation between individual PP showed that each PP becomes colonized by a unique set of WITS. This indicates that the invasive WITS pool is not shaped within the intestinal lumen. The WITS representation within pooled PP was most similar to gut draining mLN and considerably less similar to spleen and liver. This indicates that colonization of liver and spleen does not originate from GALT and/or gut draining mLN. Barnes and colleagues made a similar observation for the spread of *Y. pseudotuberculosis* [15]. In their report a set of individually tagged *Y. pseudotuberculosis* strains was generated and their presence was determined by PCR amplification and hybridization [15]. Comparing the presence or absence of tagged strains in spleen and mLN very little overlap was observed, indicating that similar to the situation for *Salmonella* described herein, for both pathogens, infection of spleen and liver does not route through gut lymphoid tissues.

The technical approach used in the *Yersinia* study did not allow determining the frequency of the tagged strains but only considered the presence or absence of a strain. Similarly, in our study we used the number of ‘missing WITS’ to estimate TSU. In contrast, however, we used NGS to avoid problems with the detection of low abundant clones and to reliably determine holes in the WITS pool. Underrepresented WITS might easily be missed by hybridization- or PCR-based approaches. Yet, in case of *S.* Typhimurium, the detection of low abundant WITS seems particularly important, because we found that WITS compositions were highly skewed. In further contrast to the *Yersinia* approach, the characterization of WITS composition by NGS allows to determine the frequency of individual WITS among the non-missing clones, which provides relevant information to compare the WITS composition between compartments. As another technical approach, quantitative PCR was used by Grant and colleagues to identify WITS after systemic infection. Minutes after systemic infection, all WITS were detectable in blood, whereas at later time points individual WITS were absent, indicating that host killing of bacteria and concomitant bacterial replication can result in the formation of subpopulations of bacteria within tissues [17]. Here we did not investigate any time point earlier than day 2 post infection and thus cannot rule out that such processes can contribute to the formation of holes in the WITS pool. Indeed the highest similarity in WITS representation was observed for the comparison of liver and spleen, an observation that is easiest explained by an early mixing of WITS between both organs.

Notably, similarity in WITS representation between liver and spleen was lost in vaccinated mice. In vaccinated mice, exchange of WITS between liver and spleen might be limited by the action of *Salmonella*-directed antibodies. In this case, the TSU observed in liver and spleen would represent the sum of bacteria seeding the individual compartments and TSU should be lower in vaccinated mice compared to naïve mice. Indeed we observed a tendency to lower TSU in the spleen of vaccinated mice compared to non-vaccinated mice. Still mixing of bacterial subpopulations generally does not seem to occur freely. In 129Sv mice that survive *S.* Typhimurium infection and become chronically infected, the WITS composition in spleen and liver was dissimilar and retained a disproportional WITS distribution similar to the situation observed at day 2 of acute infection. Comparing the TSU in PP of vaccinated and naïve mice, we observed lower TSU in the former group. This shows that vaccination reduces but does not shield PP from *Salmonella* invasion and contrast with other reports suggesting that SIg was dispensable for vaccine-mediated protection [8], [30]. Still, reduction in TSU observed in vaccinated mice is difficult to explain other than by *Salmonella*-directed SIgA. We therefore propose that experimental infections using CFU and/or clinical symptoms as readout might have missed the vaccine-mediated protection of PP. However, since infection of PP does not carry on to spleen and liver, reduced entry into PP might have only little effects with respect to colonization of spleen and liver. Similarly, there is little evidence to support a role of gut epithelial cell invasion for *Salmonella* infection *in vivo* and its overall impact to disease progression. Instead systemic compartments seem to be colonized by a low number of bacteria presumably directly entering the blood circulation within gut tissues.

In conclusion, we have established and validated a NGS-based method to describe *Salmonella* population dynamics. NGS offers a robust method to characterize WITS compositions and in contrast to other methods benefits from the high resolution offered by a complex WITS library. We propose that the technical approach described in this study may help the characterization of critical steps during bacterial infection and spur the development of new anti-infective therapies. We show that infection in PP fuels spread to the mLN but not liver and spleen. Vaccination uncoupled colonization of liver and spleen and moderately reduced colonization of PP, a result that can best be explained by the presence of *Salmonella*-directed SIg in orally vaccinated mice.

## Materials and Methods

### Ethics statement

Experiments involving animals were performed in accordance with the German Law for the Protection of Animal Welfare (Tierschutzgesetz). Experiments were approved by the Lower saxony state office for consumer protection and food safety (Landesamt für Verbraucherschutz und Lebensmittelsicherheit, LAVES) under the file numbers TVA 09/1771, TVA 10/0164 and TVA 12/0915.

### Bacterial strains

*Salmonella* strains were derivatives of *S. enterica* serovar Typhimurium strain SL1344. SL1344Δ*aro*A and SL1344Δ*hil*A (SPI-1 deficient) were described previously [22], [31]. Fluorescence-tagged variants were generated by transforming plasmid pBAD18, containing the desired fluorochrome gene (dsRed, GFP or mPlum) under control of an arabinose inducible promoter, by electroporation. A 23-clones library of nucleotide-tagged *S.* Typhimurium SL1344 was established. Each clone was generated by integrating of a PCR product into the *proV* gene in the *Salmonella* chromosomal genome. The PCR product contains a degenerate four nucleotide sequence and a chloramphenicol resistance cassette (amplified from the pKD3-plasmid), flanked by parts of the *proV* gene. As described previously [32], integration was performed with the aid of a phage λ Red recombinase-containing plasmid pKD46 which was already present in the parental *Salmonella* strain. Each clone differed from one another by only 4 bp in the nucleotide tag gene sequence. To select suitable clones for the WITS library, individual colonies were picked, the tagged site amplified by PCR and sequenced. Clones to be included in the library were selected for easy to identify tag sequences with sufficient differences to avoid erroneous assignment of tags. In particular we avoided tag sequences, which by single nucleotide differences/sequencing errors can be interconverted.

### Bacteria culture, immunization and infection

*S.* Typhimurium were grown in LB broth, until the culture had reached a density of OD_600_1.0–1.2. Bacteria were washed with 3% NaHCO_3_/LB medium, and resuspended to final cell density of 1×10^9^ bacteria (SL1344 parental strain, Δ*aro*A and Δ*hil*A) or 2.3×10^9^ bacteria (nucleotide-tagged *Salmonella*, each clone is represented by 10^8^ bacteria) per 100 µl NaHCO_3_/LB. Mice were infected by oral gavage with 100 µl bacterial suspension for 2 days (acute infection experiment) or immunized orally with 1×10^9^ live attenuated SL1344 Δ*aro*A for at least 40 days. Vaccinated mice were given one dose of enrofloxacin (Baytril, Bayer, Leverkusen) orally at 2.5 mg in 100 µl PBS to clear any remaining *Salmonella* in the lumen, 2 days before the mice were re-infected orally with 1×10^9^ CFU of SL1344. The actual numbers of inoculated bacteria and of bacteria recovered from tissues were determined by serial dilution plating on selective LB agar plates with appropriate antibiotics (90 µg of streptomycin/ml or/and 100 µg of ampicillin/ml). Organs were dissected and homogenized with Ultra Turrax T18 (Carl Roth, Karlsruhe). Aliquots from these homogenized tissue samples or the whole cell suspensions, were first mixed and vortexed with 3% Triton X-100 (Sigma-Aldrich), then plated on selective LB agar plates and incubated overnight at 37°C.For fluorescence-tagged *Salmonella* clones colonies were counted under a fluorescence stereomicroscope (Leica MZ16FA).

### Genomic DNA extraction, PCR and next generation sequencing of nucleotide-tagged *Salmonella* library clones

Colonies were washed off in 4 ml PBS on a horizontal shaker. Bacteria suspensions were collected, centrifuged and pellets resuspended. For genomic DNA extraction 4 µl of the cell pellet was transferred to 1 ml of 0.1% SDS, 10 mM Tris HCL, 5 mM EDTA, and incubated at 60°C for 30 minutes to lyse the cells. Genomic DNA was extracted from each sample, by the conventional ‘Phenol-chloroform’ DNA isolation method.

Ten ng of gDNA from each sample was used as template for a two step PCR reaction creating an amplicon library fully compatible to the multiplexing Illumina TruSeq DNA sequencing protocol (size of first product = 138 bases, inclusive of the target sequence 70 bases: ACAGGACGAAGACCGTGAATATGGTTACGTCATTGAGCNNNNTGTGTAGGCTGGAGCTGCTTCGAAGTTC; Forward adapter primer sequence: ACACTCTTTCCCTACACGACGCTCTTCCGATCTACAGGACGAAGACCGTGAATATGG; Reverse adapter primer sequence: GTGACTGGAGTTCAGACGTGTGCTCTTCCGATCTGAACTTCGAAGCAGCTCCAG).

The second step PCR was performed to add the multiplex tag (MID) assigned to each sample, while it extended the final PCR product to 193 base pairs in total length (Forward multiplex adapter primer: AATGATACGGCGACCACCGAGATCTACACTCTTTCCCTAC; Reverse multiplex adaptor primer: CAAGCAGAAGACGGCATACGAGATNNNNNNGTGACTGGAGTTCAGAC. Underlines indicate MIDs).

QIAquickGel extraction kit (Qiagen) was used to extract and purify the PCR amplicons from the gel according to the manufacturer's instruction. The concentration of the final PCR amplicon library was adjusted and sequenced on Illumina a MiSeq system using MiSeq Reagent Kits (73cycles/323cycles) at the Genome Analytics Group (GMAK) in Brunswick, Germany. The fluorescent images were processed to sequences and transformed to FastQ format using the Real Time Analysis Software RTA 1.17.22 (Illumina). Low quality reads were discarded. Sequences were further processed with in house Microsoft Excel VBA (Visual Basic for Applications) based macros. Only sequences that comprised intact flanking regions up- and downstream of the nucleotide tag were included in the analysis and the frequency of every individual nucleotide tag was determined. Tag-sequences not represented in the WITS library are likely to be a consequence of sequencing errors and were discarded.

### Ablation of CD11c^+^ cells

CD11c-DOG mice [33] were injected intraperitoneally with diphtheria toxin (DT) at 1 µg per mouse 18 hours before *Salmonella* infection. The efficacy of DT-induced CD11c^+^ DC depletion in PP, mLN and spleen was confirmed by flow cytometry (data not shown).

### Mice

Mice were bred at the central animal facility of Hannover Medical School under specified pathogen-free conditions. C57BL/6 mice and 129Sv mice were purchased from the Charles River Laboratory (Sulzfeld, Germany).

### Statistical analysis

TSU were determined by a custom made Microsoft Excel VBA (Visual Basic for Applications) based macro (Text S1) and provided as supplementary material. MHI was analysed with *BiodivR* 1.2 software (Hardy, O.J. 2010. *BiodivR* 1.2. A program using rarefaction principles was applied to compute statistically unbiased indices of species diversity within sample and species similarity between samples (http://ebe.ulb.ac.be/ebe/Software.html). MHI = 0 indicates that 2 samples do not overlap at all in terms of clonal representation, whereas MHI = 1 implies that both samples share the same set of clones and in similar proportions for each clone. Statistical analysis was performed with GraphPad Prism software. Unpaired nonparametric two-tailed *t* test (Mann-Whitney test) was used to determine inter-group significant values. Whisker bar lines represent 95% confidence interval range. Statistical differences of the mean values are indicated as follows: *, *P* value is <0.05; **, *P* value is <0.01; and ***, *P* value is <0.001.

## Supporting Information

Figure S1 **Nucleotide-tagging does not affect** ***S.*** **Typhimurium virulence.** Mice were infected with a 1∶1 mixture of SL1344 (open boxes) and a WITS library clone (closed circles). Two days after oral infection, CFU were determined for each strain by differential plating of minced mLN and single PP or five days after intra peritoneal infection CFU were determined in liver and spleen (spl). Symbols indicate individual organs and bold lines median CFU from 2 pooled experiments. Numbers indicate organs displaying less than 10 CFU.
(TIF)

Figure S2 **WITS did not acquire stable virulence-affecting modifications** ***in vivo*** **.** Two 129Sv mice were infected with 10 WITS strains and 60 days after infection the contribution of each strain determined for the mLN. WITS clone 5 has intentionally been excluded because this clone did not grow well in this particular experiment. For each mouse a pair of one over- and one underrepresented WITS was picked (marked by asterisks) and used for a second round of oral infection using both WITS at equal proportions. Similar, WITS compositions were observed in 4 additional mice analysed in 2 independent experiments. After 2 days the mice were sacrificed and the contribution of both strains to the pool of *Salmonella* in the mLN determined. Numbers indicate the number of colonies analysed.
(TIF)

Figure S3 **PCR amplification and NGS results in highly reproducible results.** The identical sample was used for independent isolation of gDNA, PCR amplification and sequencing. The plot illustrates the frequency of each WITS in the independent technical replicates. Symbols indicate individual organs pooled from 7 independent experiments.
(TIF)

Figure S4 **Qualitative assessment of WITS distribution in different compartments.** Scatter plots based on WITS frequencies between compartments in (A) non-vaccinated mice and (B) mice vaccinated with attenuated *Salmonella* 40–50 days before. Numbers in the lower left corner of the diagram indicate the WITS undetectable in both compartments depicted in the respective diagram.
(TIF)

Figure S5 **DC contribute to dissemination of** ***Salmonella*** **to mLN but not systemic compartments.** To deplete DC, mice expressing the diphtheria toxin receptor under control of the CD11c promoter [33] were injected with Diphtheria toxin 18 hours before oral challenge with a mixture of 23 WITS. (A) TSU and (B) CFU were determined for mLN, liver and spleen, at 2 days post infection. Middle lines indicate either mean TSU or median CFU. **p<0.01, ***p<0.001.
(TIF)

Text S1 **Provides a brief description of the VBA-coded simulation to estimate TSU.**
(DOCX)
