# Supplementary figures and images for: Independent Bottlenecks Characterize Colonization of Systemic Compartments and Gut Lymphoid Tissue by Salmonella
Source: PLoS Pathog. 2014 Jul 31;10(7):e1004270. doi: 10.1371/journal.ppat.1004270 (PMC4117638; doi:10.1371/journal.ppat.1004270)

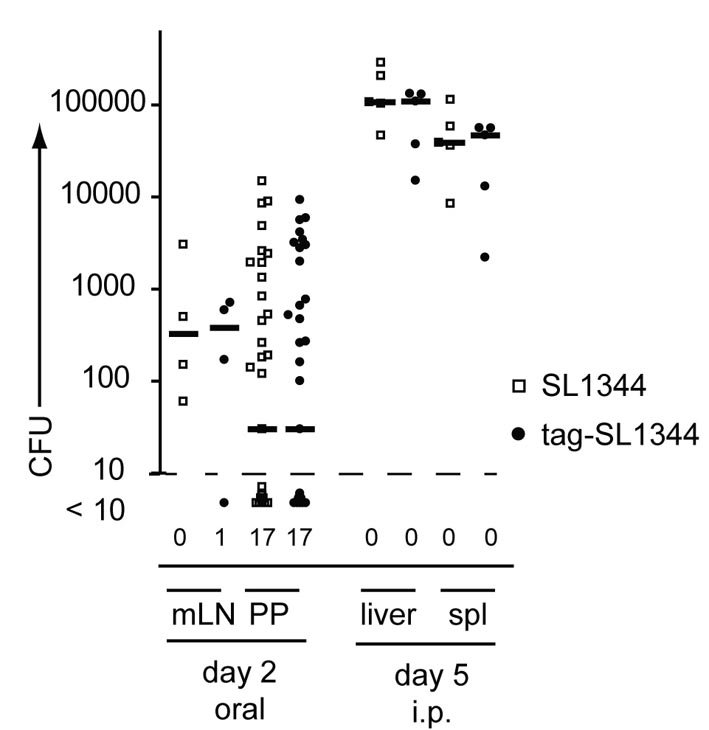

Supplement: Figure S1 — Nucleotide-tagging does not affect S. Typhimurium virulence. Mice were infected with a 1∶1 mixture of SL1344 (open boxes) and a WITS library clone (closed circles). Two days after oral infection, CFU were determined for each strain by differential plating of minced mLN and single PP or five days after intra peritoneal infection CFU were determined in liver and spleen (spl). Symbols indicate individual organs and bold lines median CFU from 2 pooled experiments. Numbers indicate organs displaying less than 10 CFU. (TIF) [file ppat.1004270.s001.tif]

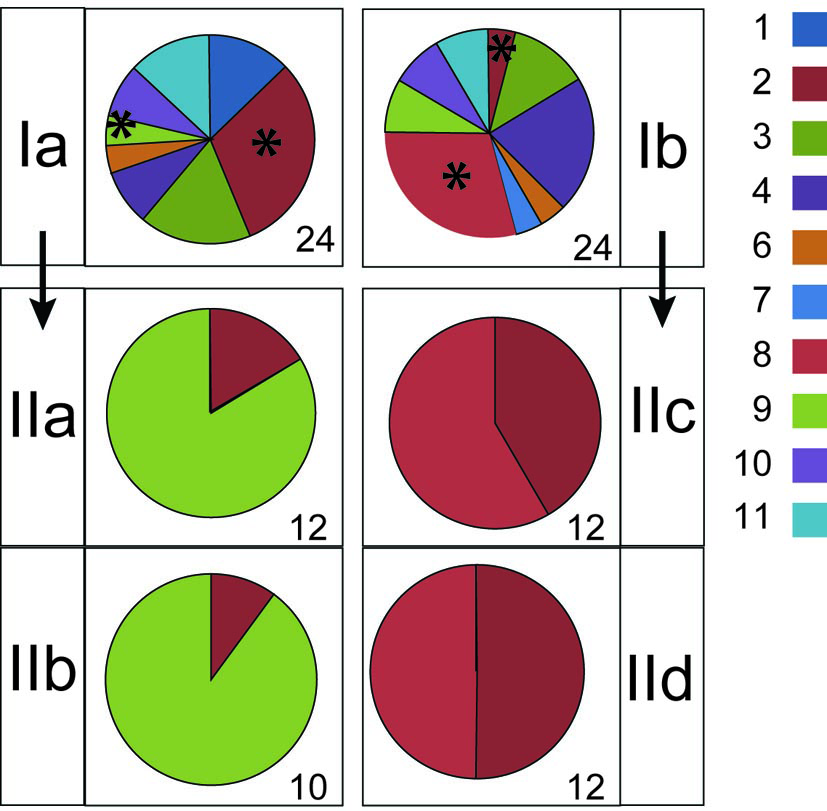

Supplement: Figure S2 — WITS did not acquire stable virulence-affecting modifications in vivo . Two 129Sv mice were infected with 10 WITS strains and 60 days after infection the contribution of each strain determined for the mLN. WITS clone 5 has intentionally been excluded because this clone did not grow well in this particular experiment. For each mouse a pair of one over- and one underrepresented WITS was picked (marked by asterisks) and used for a second round of oral infection using both WITS at equal proportions. Similar, WITS compositions were observed in 4 additional mice analysed in 2 independent experiments. After 2 days the mice were sacrificed and the contribution of both strains to the pool of Salmonella in the mLN determined. Numbers indicate the number of colonies analysed. (TIF) [file ppat.1004270.s002.tif]

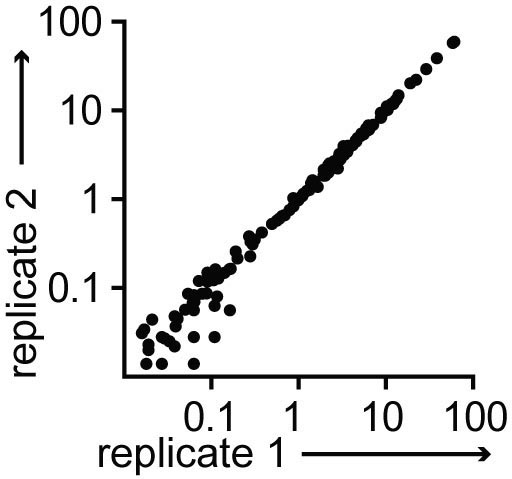

Supplement: Figure S3 — PCR amplification and NGS results in highly reproducible results. The identical sample was used for independent isolation of gDNA, PCR amplification and sequencing. The plot illustrates the frequency of each WITS in the independent technical replicates. Symbols indicate individual organs pooled from 7 independent experiments. (TIF) [file ppat.1004270.s003.tif]

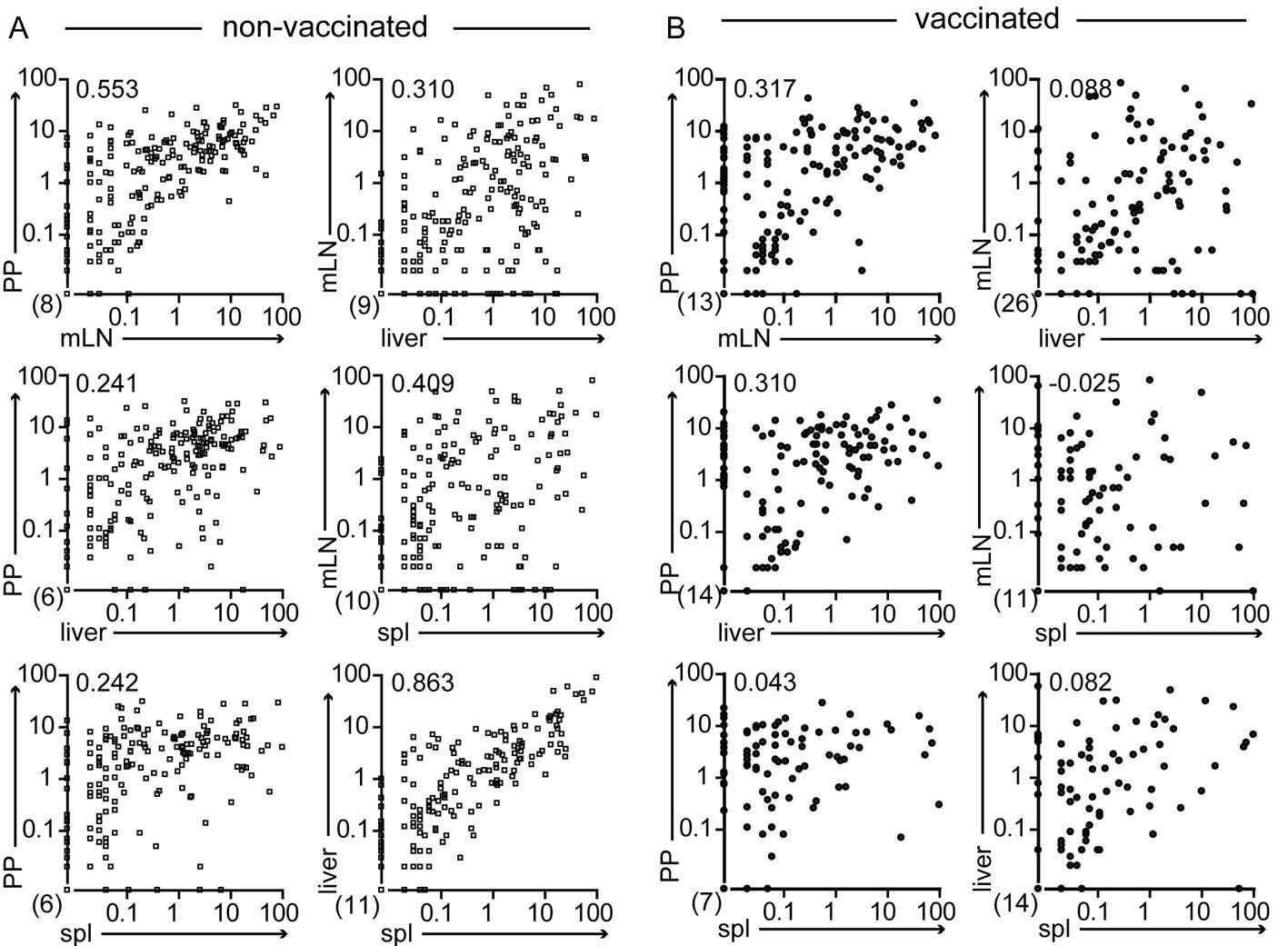

Supplement: Figure S4 — Qualitative assessment of WITS distribution in different compartments. Scatter plots based on WITS frequencies between compartments in (A) non-vaccinated mice and (B) mice vaccinated with attenuated Salmonella 40–50 days before. Numbers in the lower left corner of the diagram indicate the WITS undetectable in both compartments depicted in the respective diagram. (TIF) [file ppat.1004270.s004.tif]

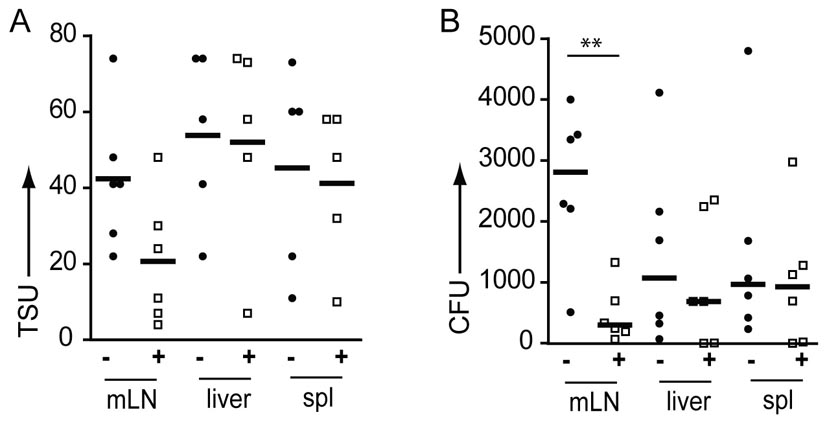

Supplement: Figure S5 — DC contribute to dissemination of Salmonella to mLN but not systemic compartments. To deplete DC, mice expressing the diphtheria toxin receptor under control of the CD11c promoter [33] were injected with Diphtheria toxin 18 hours before oral challenge with a mixture of 23 WITS. (A) TSU and (B) CFU were determined for mLN, liver and spleen, at 2 days post infection. Middle lines indicate either mean TSU or median CFU. **p<0.01, ***p<0.001. (TIF) [file ppat.1004270.s005.tif]
