# Supplementary material for: Independent Bottlenecks Characterize Colonization of Systemic Compartments and Gut Lymphoid Tissue by Salmonella
Source: PLoS Pathog. 2014 Jul 31;10(7):e1004270. doi: 10.1371/journal.ppat.1004270 (PMC4117638; doi:10.1371/journal.ppat.1004270)
Supplement: Text S1 — Provides a brief description of the VBA-coded simulation to estimate TSU. (DOCX) [file ppat.1004270.s006.docx]

**Text S1**

*In silico* simulation of invasion

In this study we used *in silico* simulation to translate experimentally observed frequencies of ´WITS missing´ into TSU, i.e. the most likely number of bacteria founding the infection. The *in silico* simulation offers two important advantages compared to a mathematical approach describing TSU as a function of ‘WITS missing’. Firstly, the simulation is highly intuitive and can easily be used and adapted by other researchers. Secondly, besides determining the theoretical TSU expected for large numbers of mice analysed, we simulate typical experiments performed on small animal groups. Thus *in silico* simulation enables us to determine confidence intervals for TSU and judge the robustness of the actual experimental observations.

The simulation is based on the following assumptions. Firstly, each WITS has an equal chance to invade, i.e. all WITS have equal virulence. The validity of this assumption has been experimentally confirmed (Fig. S1). Secondly, we assume that invasion by a given WITS does not decrease its chance for further invasion. This second assumption is justified by the large number of bacteria typically used for oral infection as compared to the lower number of WITS. Based on these two assumptions TSU were determined by a Microsoft Excel VBA (Visual Basic for Applications) based macro.

As input parameters we provide the number of WITS and their respective frequencies in the inoculum. This information is used to simulate random invasions that take into account the relative abundances of every WITS in the inoculum. An additional simulation parameter is the number of organs/samples analyzed. This number determines how many times every simulation cycle is repeated. Finally, we provide the experimentally determined number of ‘WITS missing’ in every organ/sample.

Every cycle of the simulation starts to assume a single invasion for every virtual organ/sample. Thereafter, the number of simulated invasions is incrementally increased, i.e. invasions by 2, 3, 4, etc. bacteria are simulated. The number of ‘WITS missing’ is determined for every virtual organ/sample and every number of simulated invasions. These results are compared to the experimental observations. If the result of the simulation matches the actual experimental observation (the number of ‘WITS missing’ in the experiment is identical to the number of ‘WITS missing’ in the simulation), the corresponding number of invasions (the TSU) is recorded. In other words, all simulated numbers of invasion that can explain the experimentally observed number of ‘WITS missing’ are stored. Until here, the simulation recapitulates a single experiment performed with the given number of organs/samples. Thereafter the entire simulation is performed repeatedly, a large number of times, generating a table of possible TSU for each organ/sample. Finally, all simulated TSU results are sorted and the median and lower and upper CI values are reported.
